# It takes two to pantomime: Communication meets motor cognition

**DOI:** 10.1016/j.nicl.2018.06.019

**Published:** 2018-06-21

**Authors:** Lisa Finkel, Katharina Hogrefe, Scott H. Frey, Georg Goldenberg, Jennifer Randerath

**Affiliations:** a Department of Psychology, University of Konstanz, Germany; b Lurija Institute for Rehabilitation Science and Health Research, Kliniken Schmieder, Allensbach, Germany; c Ludwig-Maximilians-Universität München, Germany; d Department of Psychological Sciences, University of Missouri, USA; e Technical University Munich, Germany; f Medical Practice for Cognitive Neurology, Vienna, Austria

**Keywords:** Pantomime, BPO, Motor cognition, Communication, Dual routes, Neural correlates, VOI, volumes of interest, FDR, false discovery rate, VLSM, voxelwise lesion symptom mapping

## Abstract

For over a century, pantomime of tool use has been employed to diagnose limb apraxia, a disorder of motor cognition primarily induced by left brain damage. While research consistently implicates damage to a left fronto-temporo-parietal network in limb apraxia, findings are inconsistent regarding the impact of damage to anterior versus posterior nodes within this network on pantomime. Complicating matters is the fact that tool use pantomime can be affected and evaluated at multiple levels. For instance, the production of tool use gestures requires the consideration of semantic characteristics (e.g. how to communicate the action intention) as well as motor features (e.g. forming grip and movement). Together, these factors may contribute substantially to apparent discrepancies in previously reported findings regarding neural correlates of tool use pantomime.

In the current study, 67 stroke patients with unilateral left-brain damage performed a classic pantomime task. In order to analyze different error characteristics, we evaluated the proper use of grip and movement for each pantomime. For certain objects, healthy subjects may use body parts as representative for the object, e.g. use of the fingers to indicate scissors blades. To specify the pathological use of body parts as the object (BPO) we only assessed pantomime items that were not prone to this response in healthy participants. We performed modern voxel-based lesion analyses on MRI or CT data to determine associations between brain injury and the frequency of the specific types of pantomime errors.

Our results support a model in which anterior and posterior nodes of the left fronto-temporo-parietal network contribute differentially to pantomime of tool use. More precisely, damage in the inferior frontal cortex reaching to the temporal pole is associated with an increased frequency of BPO errors, whereas damage to the inferior parietal lobe is predominantly linked to an increased frequency of movement and/or grip errors. Our work suggests that the validity of attempts to specify the neural correlates of limb apraxia based on tool use pantomime depends on differentiating the specific types of errors committed. We conclude that successful tool use pantomime involves dissociable functions with communicative aspects represented in more anterior (rather ventral) regions and motor-cognitive aspects in more posterior (rather dorsal) nodes of a left fronto-temporo-parietal network.

## Highlights

• Two core left hemisphere networks are involved in tool use pantomime.
• Pathological use of BPO strategy correlated with rather anterior lesions.
• Motor-cognitive errors associated with rather posterior lesions.
• A pathway model integrating communicative vs motor-cognitive aspects is discussed.

## 1 Introduction

The term limb apraxia refers to a motor-cognitive impairment that is characterized by impairments in planning or completing motor actions. Limb apraxic deficits often occur after stroke induced left hemisphere brain damage (e.g. Buxbaum et al., 2005; Goldenberg, 2009; Goldenberg and Randerath, 2015). For diagnosing limb apraxia, the pantomime of tool use task (often also referred to as transitive gestures) is both sensitive and widely used, along with the imitation of hand postures, and real tool use tasks (e.g. Buchmann and Randerath, 2017). Pantomiming tool use places demands on both motor-cognitive and communicative processes: it includes the simulative demonstration of a tool use action (as if the object were actually held in hand and used), and has been proposed to require the integration of semantic and motor features of the underlying tool use action (Bartolo et al., 2003; Goldenberg, 2009; Manuel et al., 2013; Niessen et al., 2014).

Several lesion studies have been conducted in attempts to determine the neural basis of the production of tool use gestures. Findings demonstrate that left inferior frontal, temporal as well as inferior parietal regions all have a prominent and also exclusive role for pantomiming tool use gestures (Buxbaum et al., 2014; Goldenberg and Randerath, 2015; Mengotti et al., 2013). However, there is a large disparity regarding the core nodes within this left fronto-temporo-parietal network, and their specific contributions to pantomime production. Some lesion studies report that damage to left brain's anterior regions is essential for impaired pantomime of tool use (i.e. inferior frontal or temporal cortices: Goldenberg et al. (2007); Manuel et al. (2013); Weiss et al. (2014)), while other studies emphasize more posterior areas (i.e. the parietal lobe and temporal regions Goldenberg and Randerath (2015); Hoeren et al. (2014); Price et al. (2010).

With respect to tool use actions, inferior frontal and temporal regions have been reported to contribute to semantic processing (Hodges et al., 2000; Hogrefe et al., 2017; Jefferies, 2013; Patterson et al., 2007) or related functional grasping movements (Randerath et al., 2010). Conversely, dorsal stream structures, including the parietal cortex, have frequently been associated with processes involving how an object or body part can be manipulated (Almeida et al., 2010; Buxbaum and Saffran, 2002; Goodale and Milner, 1992; Johnson-Frey, 2004; Tobia and Madan, 2017). These functional attributions may fit the idea of two major networks supporting pantomime of object use: one contributing predominantly to the communicative nature of pantomiming, and the other one supporting predominantly motor cognitive aspects such as the spatial configuration of the body, hands and their movements.

The communicative side of pantomiming object use concerns the depiction of objects and actions in a way that observers can understand, which is unique to pantomiming the movement without a tool compared to actual object use. Communicating object use by pantomiming can be facilitated by substituting parts of the body for the tool *(body part as object*, BPO). For example, it is easier to recognize someone demonstrating use of scissors if the pantomiming person uses two straightened opening and closing fingers resembling the blades versus the open-and-close hand movements involved in actual use of this device.

During pantomime, the motor-cognitive system also needs to cope with the absence of mechanical interaction between the hand and objects. Motor programs of actual object use need to be replicated in order to produce a movement that closely resembles real tool use movements in spatial coordinates and content. This conversion from general ideas into defined motor programs resulting in a specific movement, seem to be associated with posterior parietal regions (Buxbaum et al., 2003; Halsband et al., 2001; Weiss et al., 2008).

Thus, one significant reason for the anterior-posterior disparity of neural correlates of tool use pantomime and its ongoing discussion may be the variation in the task demands of the assessment of pantomime characteristics. We postulate that, communicative errors will be associated with damage affecting more anterior regions. By contrast, damage to more posterior regions will be associated with motor-cognitive errors.

To address this issue, the current study scored several components of the pantomimed gesture; specifically, a clear differentiation was thereby made for grip, movement, as well as BPO errors (Goodglass and Kaplan, 1963). A more recent lesion study by Manuel et al. (2013) demonstrates the importance of these distinctions. The authors examined stroke, as well as tumor patients, in a pantomime task and differentiated between typical configural/spatial and BPO pantomime error types. For their retrospective study, Manuel et al. (2013) evaluated pantomime movements from either one of two different tests consisting of 4 (Peigneux and Van der Linden, 2000) or 5 items (Mahieux-Laurent et al., 2009) respectively. They found associations of left inferior frontal lesions with both error types and additional neural correlates in temporo-parietal regions for the configural/spatial errors. Based on their data, the authors conclude that there exists a left intrahemispheric dissociation for various aspects of pantomime, but that the inferior frontal region plays a nonspecific role. The authors, however, acknowledge that their retrospective approach has limitations. Of particular relevance to the current project, no distinction was made between test items that may or may not naturally lead to BPO gestures, e.g. both tests included at least two items that are likely prone to BPO errors in healthy subjects (hammer, comb). BPO errors can and do also occur in healthy subjects (Duffy and Duffy, 1989) and as described above in specific cases, a “body part as object strategy” may help to make pantomime gestures more recognizable. One can therefore question whether such instances should be considered BPO errors when exhibited by patients.

For the current study, we consider this possibility by undertaking an a priori analysis of data from 82 healthy participants in order to differentiate between items that are prone to a “body part as object strategy” (BPO items) and items for which that strategy is uncommon (No-BPO items).

Pantomime data of 67 stroke patients with left brain damage (LBD) was also analyzed (Goldenberg et al., 2003), and modern voxel based lesion analyses (VLSM, (Bates et al., 2003)) were applied to MRI and CT data in order to determine neural correlates of grip, movement errors as well as pathologically relevant BPO errors.

With the current work, we aim to shed light on the still controversially discussed roles of inferior frontal, temporal and inferior parietal regions in pantomime production. We assume that a large part of the ongoing discussion can be explained by the chosen assessment approach and by the documented error types in a respective patient sample. Based on prior research, we hypothesized that motor-cognitive aspects depicted by movement and grip errors, are mainly associated with posterior lesions in a left fronto-temporo-parietal pantomime network. Conversely, deficits in communicative processing reflected by pathological BPO errors, were expected to be associated with lesions in rather anterior parts of this ventro-dorsal pantomime network.

## 2 Methods

A total of 117 participants (67 patients with left brain damage and 50 healthy control persons) fulfilled the inclusion criteria: i.e. right-handedness (Edinburgh Handedness Inventory (Oldfield, 1971) or hand-preference questionnaire of Salmaso and Longoni (1983)), normal or corrected-to-normal vision (at least 30 ft./9 m), being naïve to the study's hypotheses and specific goals, no other neurological or psychiatric diseases affecting cognitive abilities, and providing informed consent in accordance with the local IRB and the Declaration of Helsinki. Further, patients with history of previous stroke or an inability to understand study instructions were excluded from recruitment. Based on study objectives, patient data was only included when a brain scan was available. All participants were asked to use their left hand for the test procedure.

Patients with left unilateral infarction or hemorrhagic stroke were recruited and tested at the following locations: Hospital Bogenhausen Munich (Germany), Kliniken Schmieder in Allensbach (Germany), RUSK Rehabilitation Center in Columbia (Missouri, USA) and the University of Missouri Hospital in Columbia (Missouri, USA; study recruitment only). Recruitment and testing of healthy subjects were conducted at the University of Konstanz as well as at the RUSK Rehabilitation Center in Columbia (Missouri, US).

### 2.1 Patients

In total, 67 patients with left brain damage (LBD) fulfilled the inclusion criteria and were consequently included in the analyses. The patient group included 49% females as well as 51% males with a mean age of 56.1 years (range 26 to 82). According to the clinical charts, stroke was caused by infarction in 66% and by hemorrhage in 33% of cases. For one patient, the clinical chart was not clearly documented so that a clear assignment proves to be difficult. All patients were tested within a range of 3 weeks post stroke up to 116 months (M = 13.31, SD = 21.11; in months). All stroke patients used their ipsilesional left hand whilst testing. 63% of the patients were diagnosed with aphasia based on clinical reports and 73% of LBD patients were diagnosed with limb apraxia based on the evaluation of pantomime performance (see Table 1 for an overview of descriptives and supplementary material Appendix A, Table 1 for raw scores).

**Table 1:** Descriptive data for healthy controls and LBD patients.

	Healthy controls (N = 50)	LBD patients (N = 67)
Age		
Mean	52.6	56.1
SD	16.7	13.8
Sex		
Male (N)	23	34
Female (N)	27	33
Months post stroke	–	
Mean		13.3
SD		21.1
Stroke etiology	–	
Infarction (N)		44
Hemorrhage (N)		22
Not clearly documented (N)		1
Apraxia^a^	–	
Yes (N)		49
No (N)		18
Aphasia^b^	–	
Yes (N)		42
No (N)		25

a Based on total pantomime score (13 items). Cut-Off was set at the 5th percentile of the healthy control group.

b Based on clinical reports

### 2.2 Healthy controls

We included 50 neurologically and psychiatrically healthy control subjects with a mean age of 52.6 years (range 21 to 79). Healthy controls were matched to the stroke patient group in age level (U = 1548.0, p = .389). To ensure that there is a sufficiently high level of general cognitive functioning, all of them had a 3MS-R (revised version of the Mini Mental State Exam) score larger than 88 (Alexopoulos et al., 2007; Tschanz et al., 2002).

## 3 Materials

All participants were tested in the production of tool use pantomime gestures (Goldenberg et al., 2003). Due to several testing locations and the usage of three versions of the Goldenberg pantomime task, test forms slightly differed concerning their length. For analysis, 13 consistent items of the three pantomime test versions were evaluated. Test instructions, test evaluation and documentation did not differ between test versions. These were based on the methodical procedure as described by Goldenberg (2011). An independent rater, who was trained to rate apraxic behavior, evaluated the entire dataset (videos) from the different sites. Adequate interrater reliability for the assessment of pantomime of tool use has been reported for example by Randerath et al. (2017).

### 3.1 Pantomime task

Subjects were asked to mime the use of 13 common objects (pounding a nail with a hammer, drinking from a glass, writing with a pencil, ironing with a flat‑iron, combing with a comb, stirring coffee with a spoon, looking through binoculars, watering with a watering can, cutting with scissors, opening a padlock with a key, brushing teeth with a toothbrush, screwing in an electric bulb, sawing with a saw). The examiner named the action as well as the object and simultaneously showed a picture of the object (e.g. can you show me how to drink from a glass). The picture of the current object was kept in view for the entire time whilst participants pantomimed the object's use. Practice items were included, and task comprehension was assumed when subjects at least attempted to produce a meaningful gesture, i.e. produced movements that were clearly different from beats synchronized with a stream of verbal utterances, from manual actions directed towards the presented picture, or from attempts to draw the outlines of the tool with the finger upon the table.

### 3.2 Item classification

In order to ascertain whether errors are really pathologically relevant, it was differentiated between items that are vulnerable for BPO errors even in healthy subjects and items with low susceptibility to such errors. For categorizing items into BPO vulnerable and BPO resistant, we used the error frequencies per item made by a larger normative sample of 82 healthy subjects included in previous work (Buchmann and Randerath, 2017; Randerath et al., 2017). Items were categorized as BPO items if one or more errors occurred when healthy subjects performed the pantomime task. In the case of a single error occurrence, this error had to persist when the person was asked to try again one more time. In contrast, items were defined as No-BPO items if no healthy subject produced an error or if a single error occurred only once and did not persist for retrial. Accordingly, typical objects for BPO errors were *glass*, *pencil*, *comb*, *binoculars, hammer* and *scissors*, whereas *iron*, *watering can*, *key*, *toothbrush*, *bulb, spoon* and *saw* were defined as No-BPO items.

In order to focus on the pathological use of BPO in our patient sample, we only present results for 7 items that were classified as No-BPO items. For the sake of completeness, VLSM analyses for the performed pantomimes using the six BPO items are added to the supplementary material (Appendix B).

### 3.3 Scoring

In the following error evaluation, we differentiate between a *classic pantomime score*, *motor-cognitive errors* (movement and grip errors) and *BPO errors*. In general the following applies: the higher the score the better the pantomime production.

#### 3.3.1 Classic pantomime score

As described in the Goldenberg pantomime test (Goldenberg, 2011), each item was rated by judging the presence or absence of predefined features of the particular pantomime concerning grip and movement. For some items space was additionally considered (e.g. writing with a pencil (3 points in total): Pincer grip directed to the table (grip: 1 point); repetitive, small amplitude movement, executed parallel to the table (movement: 1 point); distance of fingers to the table (space: 1 point)). The total score thus provides information about the subjects' general ability to produce transitive pantomime gestures. In the classic test for diagnosing impairments in pantomime production, all items were evaluated for movement and grip, and for a few items the parameter space was also assessed.

For the extracted seven NoBPO-items, the total pantomime score (maximum 17 points) included separate scores for the assessment of appropriate grip formation (maximum 7 points), movement production (maximum 7 points) and consideration of space (maximum 3 points).

For our study purposes we further only concentrated on the parameters that were assessed throughout all items. Thus, as specified below, we focused on motor-cognitive errors including configural aspects in the pantomime production of movement and grip. In addition we evaluated for all items whether subjects erroneously used a BPO strategy. For our scale of interest with seven NoBPO-Items a maximum score of seven points for each of the three variables could be achieved. Conversely this also means, there is a maximum of 7 errors per variable. Please note, because only three of the seven items were evaluated based on predefined space characteristics of the pantomime, a separate space variable was not further evaluated.

#### 3.3.2 Motor-cognitive errors

We classified incorrect grips and incorrect or incomplete movements as motor-cognitive errors. Please note that the use of a BPO strategy (e.g. using one finger as if it was a key) was not counted as grip error despite the deviation from set grip-criteria that include forming fingers as if the object would be held in hand (e.g. pincer or lateral grip for holding a key).

#### 3.3.3 Erroneous use of BPO strategy

Per item any use of body parts as the object itself was noted, irrespective of whether or not the BPO strategy persisted when the person was asked to try again one more time.

To enable statements about specific error frequencies, percentage values were calculated.

### 3.4 Additional descriptive information

We further report the presence of aphasia, neglect and hemiparesis which was determined by diagnostic material that differed in content and depth at the respective sites and therefore was not used for further correlative analysis. In the Hospital Bogenhausen in Munich (Germany) and in the Kliniken Schmieder in Allensbach (Germany), the AAT subtest Token Test and Naming were applied (Huber et al., 1983). At the RUSK Rehabilitation Center in Columbia (Missouri, US), we confirmed the information retrieved by the clinic's patient record by extracting language function from the Mini Mental State Exam (writing and naming body parts).

To report the presence of neglect and hemiparesis, we applied the subtests line bisection and star cancellation of the Behavioral Inattention Test (Wilson et al., 1987) as well as the Wolf Motor Function Test (Wolf et al., 2001) at the RUSK Rehabilitation Hospital as well as at the Kliniken Schmieder in Allensbach. At the Hospital Bogenhausen in Munich the information on the presence of neglect and hemiparesis was retrieved from the patient record. Based on this information in the current sample 81% were hemiparetic, 63% aphasic and for 22% neglect was reported.

## 4 Behavioral data analysis

Prior to our lesion data analysis, we conducted a behavioral data analysis in order to determine potential influential factors to be taken into account for lesion data analysis.

Since scores and error rates in No-BPO items appeared not to be normally distributed in both groups (C: Chi-Square ≥ 25.92, p < .001, LBD: Chi-Square ≥ 76.86, p < .001) behavioral data were analyzed non-parametrically with SPSS 24 (IBM). We generally report significances 2-tailed (p < .05) and p-values were reported exact instead of asymptotic, whenever computing power was sufficient.

### 4.1 Within group comparison

We first confirmed by within group comparison (Wilcoxon) that our item classification into a set of BPO vulnerable and BPO resistant items also holds for the tested patient sample.

### 4.2 Correlations

To examine whether there is a linear relationship between certain variables, correlations were calculated by Kendall's Tau. Possible correlations between occurring error types were analyzed in order to consider interrelations for further statistical analyses.

### 4.3 Between group comparisons

Regarding the fact, that stroke often occurs at a mature age, it seemed important to clarify whether error frequency is generally affected by age. To analyze possible effects of age in our healthy controls sample, error comparisons were run between the 25% of the youngest (range of 21 to 40 years) and 25% of the oldest participants (range of 64 to 79 years).

Additionally, we analyzed whether there are effects of age or time since the incident, which if present should be taken into account for subsequent analyses. We separated patients into acute-subacute (1–3 months) and subacute-chronic (>3 months) and tested for group differences.

## 5 Lesion data analysis

We used a voxelwise lesion symptom mapping (VLSM) approach to determine neuronal correlates of motor-cognitive errors, distinguishing between grip and movement characteristics of the pantomime, as well as BPO errors. We therefore manually or semi-automatically delineated lesions from MRI or CT-Scans. If multiple images with same quality were available, the first recorded scan was used for the analyses.

### 5.1 Lesion delineation

Lesions were delineated using two different lesion mapping methods. One part of lesions (N = 23) was manually mapped by a neurologist on an 8 mm template using MRIcro (for similar procedure see Randerath et al., 2010) including an anatomical brain atlas (Kretschmann and Weinrich, 2007) as well as the Automatic Anatomical Labeling template (Tzourio-Mazoyer et al., 2002) implemented in MRIcron for determining lesion locus. For manual lesion delineation adequate interrater reliability has been reported by Randerath et al. (2010). The other part of lesions was semi-automatically demarcated by another experimenter using Clusterize Toolbox for SPM8 (Clas et al., 2012; de Haan et al., 2015). To verify that the spatial position of the resulting Volumes of Interest (VOIs) is adequate, brain maps were further checked by comparing the lesions on everyone's structural scan with the VOI displayed on a one millimeter template (ch2bet). Where adjusting was required, lesion maps were manually corrected with MRIcron Software (Rorden and Brett, 2000). Both examiners were naïve to the clinical profiles of the patients at the time of lesion mapping.

### 5.2 Statistical analysis of neural correlates

With the aim of identifying critical brain regions that are associated with deficits in the pantomime task, we analyzed brain imaging data by using the *t*-test for continuous data in the Non-Parametric Mapping software (available with the MRIcron software). We only tested voxels that are damaged in at least 5% of patients.

Results were reported below a false discovery rate (FDR) corrected statistical threshold (p < .05) and were mapped on the ch2 template (distributed with the MRIcron software). Lesion locus was determined by both an atlas (Kretschmann and Weinrich, 2007; Petrides, 2012) and the Automated Anatomical Labelling (AAL) template provided by MRIcron.

Since behavioral analyses revealed a correlation between movement errors and pathological BPO use, we considered BPO use as covariate. For the VLSM analysis of movement errors we consequently utilized residuals as behavioral correlate.

Please note that since BPO errors overall occurred comparatively seldom compared to movement errors, analyses for BPO errors considering movement as covariate did not survive FDR correction because of insufficient statistical power. However, in order to support the specificity found in the statistical results, we ran a post hoc subtraction analysis.

### 5.3 Post hoc subtraction analysis of neural correlates

Providing further insight into neural correlates of specific error types, we additionally conducted voxel based subtraction analyses. For this we used data of only those patients demonstrating nearly selective deficits. For each error group (either grip errors, or movement errors, or BPO use) 4 patients committed more than one error in exclusively one group of error type. To run a voxel based subtraction analysis we first built overlays for each specific error group. Subsequently error type overlays were subtracted from each other. The resulting subtraction maps were mapped onto the ch2 template (distributed with the MRIcron software). This type of post hoc subtraction approach is useful to contrast selective deficits (for either motor-cognitive or communicative aspects of the pantomime task) and at the same time it helps to distinguish the associated functional regions from those that are in general frequently damaged after unilateral stroke (Rorden and Karnath, 2004).

## 6 Results

### 6.1 Behavioral results

Similar to our reference sample of healthy controls (C: Z = −3.246, p < .001), data analyses in LBD patients confirmed that the BPO error frequency in BPO items was higher than in No-BPO items (Z = −4.886, p < .001). For the sake of completeness, descriptive data is reported for both item types (BPO items in grey). However, in order to focus on the pathological use of BPO in our patient sample, we hereafter only present statistical results for items classified as No-BPO items. Error-rates in healthy controls are generally low. Accordingly, the behavioral data analyses for No-BPO items in the group of healthy participants showed no significant differences in error rates for BPO and motor-cognitive errors (grip errors (%): Z = −0.816, p = .688; movement errors (%): Z = −1.960, p = .07). In contrast, the occurrence of either type of motor-cognitive error was more frequent than BPO errors in LBD patients (grip errors (%): Z = −3.251 p < .001; movement errors (%): Z = −2.789, p = .004). These data are summarized in Table 2.

**Table 2:**
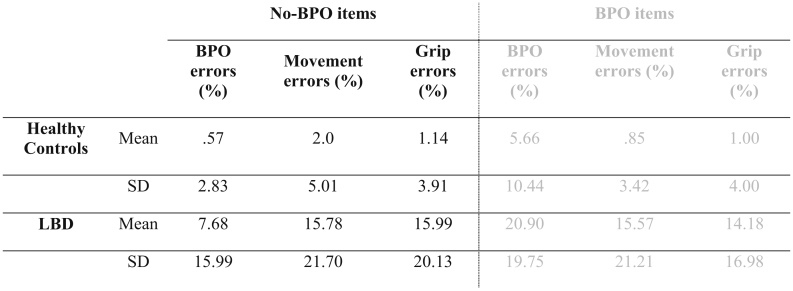
Descriptive data for error types in No-BPO and BPO items. The latter are displayed in grey, since following statistical analysis for this study focus on No-BPO items only.

A comparison of healthy young (21–40 years) and elderly (64–79) groups, failed to reveal any significant age effects on error frequency. Likewise, correlations between age and error frequency (*r* ≤ −0.024 p > .114) across all 50 healthy controls failed to achieve significance.

Similarly, the comparison of pantomime performance between acute-subacute and subacute-chronic patients showed no significant differences for either error type (grip errors (%): U = 498.0 p = .480, movement errors (%): U = 497.0, p = .468, BPO errors (%): U = 456.0, p = .138), suggesting that inter-individual differences in time since the incidence did not crucially influence task performance. This is also reflected by the low correlation coefficients between scores and time since stroke onset (*r* ≤ 0.074, p ≥ .461).

#### 6.1.1 Effects of stroke

Patients made more motor-cognitive errors (grip errors (%): U = 777.0, p < .001; movement errors (%): U = 975.0, p < .001) and more BPO errors (U = 1233.0, p < .001) in the pantomime task than healthy controls.

#### 6.1.2 Correlations between error types

There was no significant correlation between grip and BPO errors (*r* = 0.120, p = .271) in the patient group. Because movement error types correlated with BPO errors (*r* = 0.262, p = .016), however, it is assumed that these two variables may interfere with each other. For this reason, we considered BPO errors as a covariate for movement errors, and our main VLSM analysis in the next section we used movement error residuals. This made it possible to examine whether there actually are differences between movement and BPO errors with respect to lesion location.

### 6.2 Lesion analysis results

**Fig. 1:**
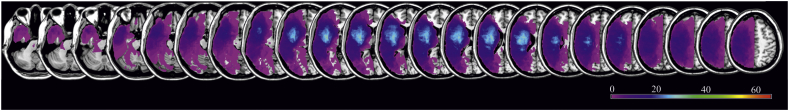
Lesion overlay. Overlay of LBD patients' lesion maps. The color bar indicates degree of overlap of lesions out of 67 patients.

Highest lesion density sat in the territory of the left middle cerebral artery (Fig. 1) and included the insula, basal ganglia as well as pre- and post-central gyrus.

Because the apparent functional significance of locations associated with larger lesions is generally diminished by lesion volume corrections (Karnath et al., 2004), we here refrained from applying this adjustment. Higher frequencies in grip errors (*r* = 0.196, p = .036) as well as movement errors (*r* = 0.291, p = .002) correlated significantly with larger lesion volumes. A similar trend was observed between BPO errors and lesion volumes (*r* = 0.166, p = .087).

#### 6.2.1 Statistical analysis of neural correlates

**Fig. 2:**
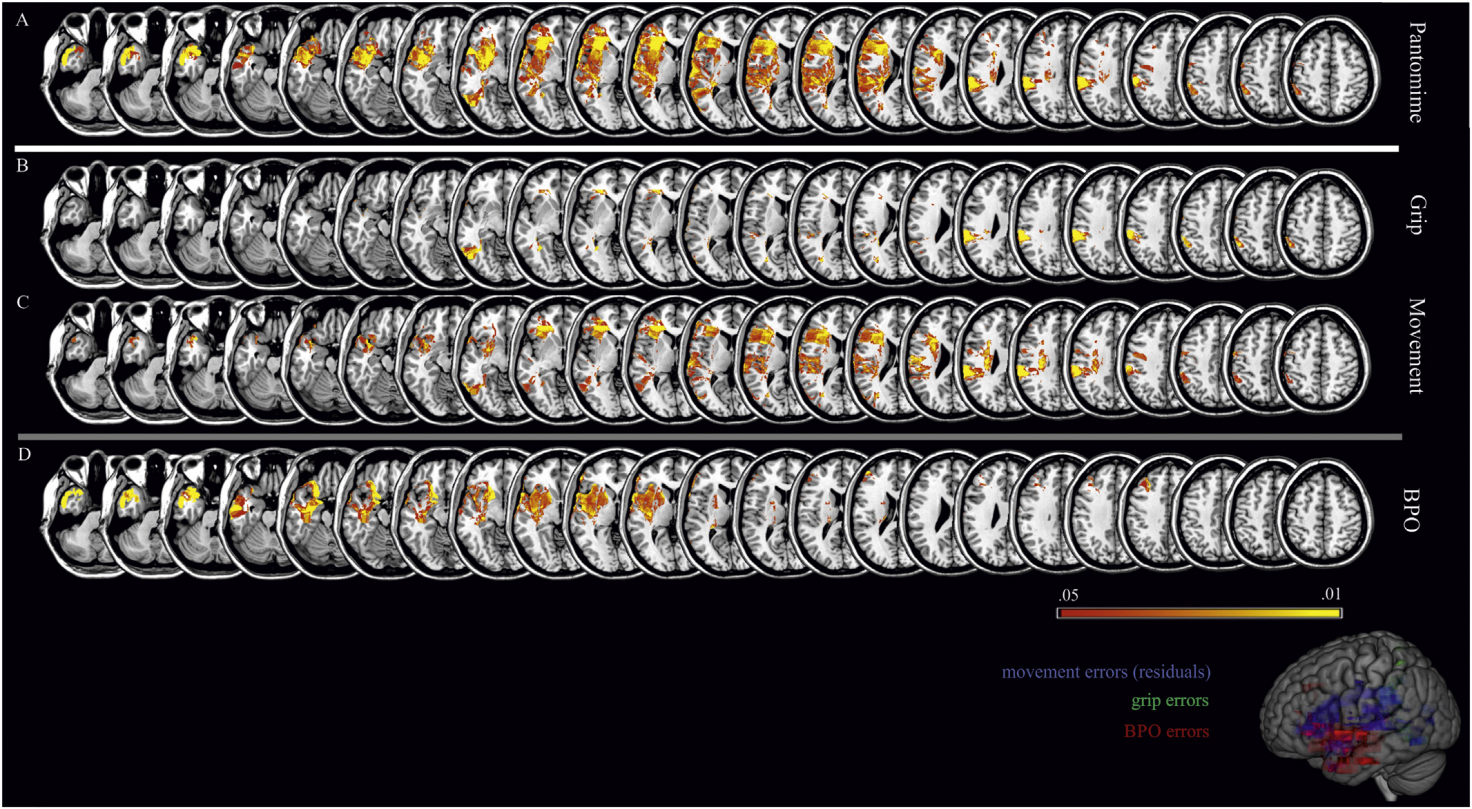
VLSM analyses for error types limited to No-BPO items (*t*-test values, FDR corrected with p < .05). First map depicts lesion locations that are related with an impaired pantomime production (total pantomime score) (A). Following maps display voxels that are either associated with motor-cognitive errors in pantomime production (grip (B) and movement residuals (C)) or communicative errors (BPO errors (D)). Critical lesion areas associated with movement (blue), grip (green) and BPO errors (red) are additionally displayed in the sagittal view.

**Fig. 3:**
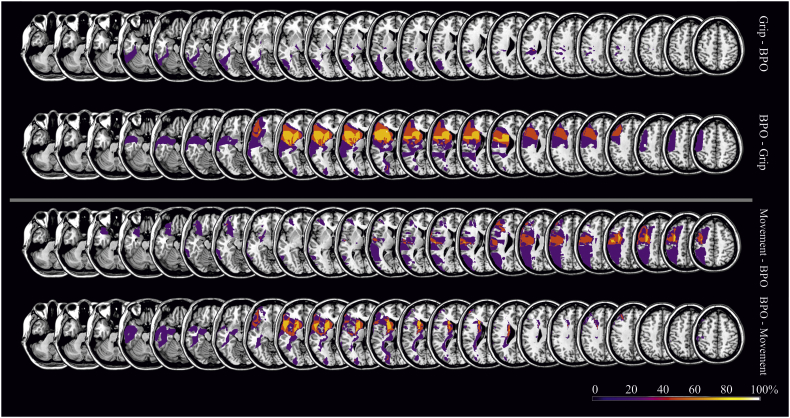
Subtraction analyses. Subtraction analyses are based on lesion overlaps of patients that committed more than one error in exclusively one group of error type (either grip, or movement, or BPO errors; overlays consist of 4 patients each error group). Colored areas display percentage of overlapping regions which are damaged in patients selectively produced one type of error.

We investigated predominant associations between diminished performance in pantomime in general (total score) as well as in specific aspects of pantomiming (grip, movement and body parts as the object errors) with critical lesion areas by means of a VLSM analyses based on 67 patient scans (Fig. 2). Due to significant correlations between movement and BPO errors, we displayed VLSM maps with BPO errors as covariate for movement errors (Fig. 2C). Post hoc, we confirmed our statistical approach by using a more conservative subtraction analysis, separately demonstrating neural correlates for motor-cognitive and BPO errors (see Fig. 3).

As can be seen in Fig. 2A, the current findings replicated previous results concerning the contribution of lesions in inferior frontal, temporal as well as inferior parietal regions to general impaired pantomime production. Moreover, Fig. 2B shows that the impairment in grip formation was mainly associated with lesions involving the supramarginal gyrus and inferior parietal regions. Movement errors in pantomime production (Fig. 2C) were linked to lesions in an inferior fronto-parietal network including the inferior frontal lobe and the inferior parietal lobe (particularly the supramarginal gyrus). Albeit less striking, the inferior and middle temporal regions showed spots associated with movement errors.

Importantly, the current study showed that frequent BPO use is associated with damage to an anterior fronto-temporal network extending from inferior frontal regions to the temporal pole, as well as the basal ganglia and adjacent insula (Fig. 2D). To provide a complete picture, additional VLSM analyses were run for patient pantomime performance of the six BPO items that were determined to also elicit BPO errors in healthy adults. Results were less clear and included anterior as well as posterior regions for an increased BPO strategy use as well as grip and movement errors. Please see Supplementary fig. 1 (Appendix B) for more information.

#### 6.2.2 Post hoc subtraction analysis of neural correlates

To provide further insight into neural correlates of specific error types, we extracted data from those patients that exclusively produced just one type of error. This yielded three subgroups (grip, movement, or BPO errors) each comprised of four individuals. Subtractive comparisons between these subgroups indicated that motor-cognitive errors are associated with lesions in more posterior areas of a fronto-temporo-parietal network, whereas communicative BPO errors are associated with lesions located considerably further anterior in this network (Fig. 3).

In sum, statistical and subtraction analyses revealed a predominance of lesions in posterior fronto-parietal regions for defective grip and movement execution in pantomime production. In contrast, lesions in anterior fronto-temporal nodes of the pantomime network predicted the pathological use of BPO for pantomiming.

## 7 Discussion

Pantomiming tool use gestures is a task that is central to the diagnosis of limb apraxia. Previous studies with voxel based lesion analyses reported several regions that are essential for the generation of transitive pantomime gestures in a left lateralized, fronto-temporo-parietal network. Reports vary with respect to the predominance of either anterior or posterior lesions within this network. Pantomime is, however, a complex action involving a variety of task demands ranging from motor-cognitive to communicative, and the errors committed by apraxic patients vary accordingly. The relationship, if any, between these various types of errors and specific lesion locations is poorly understood. In an effort to address this limitation concerning the neural substrates of impaired pantomime after left hemisphere stroke, we evaluated the production of pantomimes by distinguishing the accuracy of motor-cognitive (produced movement, formed grip) versus communicative aspects (use of a body part as object strategy) of transitive pantomimes. Further, we focused on items for which healthy subjects do not use a “body part as object strategy” as a communicative aid. Our study yielded several important results and these are discussed in detail below.

### 7.1 BPO error occurrence

Behavioral results shown here, are in line with current literature and confirm that the use of a BPO strategy can also occur in healthy subjects (Duffy and Duffy, 1989). Findings for both healthy controls and stroke patients confirmed that the use of BPO for pantomiming occurs more frequently in the predefined BPO items than in the No-BPO items. Critically, however, for both sets of items a BPO strategy was significantly more prevalent in the patient group compared to healthy controls, suggesting a pathological application of this strategy.

### 7.2 Time post stroke

In this study, there were no differences between acute-subacute and subacute-chronic patients performing the pantomime task. At least in the current subject sample, these results indicate that time since stroke onset did not crucially influence task performance. With respect to recovery from apraxic deficits, this absence of spontaneous recovery suggests a poor prognosis for daily living after stroke. However, to provide a more definitive statement, a controlled, longitudinal, within subject design would be needed. By reporting at least some improvement in praxis over time, other studies provide more optimistic results (Basso et al., 2000; 1987; considering pantomime task: Stamenova et al., 2011).

### 7.3 Neural correlates

In this study, we provide additional evidence that the left inferior frontal, temporal as well as inferior parietal regions all have a prominent role in pantomiming tool use gestures. Specific components of pantomime gestures were separately judged by classifying grip and movement errors as motor-cognitive, and distinguishing them from communicative errors reflected by pathological use of BPO. This approach allowed us to differentiate lesion loci that are associated with difficulties in motor-cognitive and communicative processes.

#### 7.3.1 Motor-cognitive errors

The results of the VLSM analyses reveal a link between posterior parietal lesions for impairments in the production of motor configural aspects of pantomime gestures. Grip errors are mainly associated with parietal areas including supramarginal gyrus and inferior parietal regions, and movement errors are associated with both these parietal regions as well as inferior frontal lesions. These findings are consistent with previous findings of fMRI and lesion studies, attributing a critical role for action knowledge processing and encoding of object-related postures and movements to the left inferior parietal lobule (Canessa et al., 2008; Kalenine et al., 2010), and for accurate grasping movements and selection of the appropriate hand posture to more posterior regions in the parieto-occipital junction and inferior frontal gyrus (Grezes et al., 2003; Randerath et al., 2010).

Through associated lesions in the left supramarginal and angular gyrus, the pantomime task (particularly the motor-cognitive aspects) taps into the left ventro-dorsal processing stream, which has been proposed to be necessary for skilled, functional object-related actions (Binkofski and Buxbaum, 2013; Binkofski and Fink, 2005). The ventro-dorsal processing stream has also been called the “use system” (Binkofski and Buxbaum, 2013). Lesions in the left inferior parietal lobe repeatedly have been associated with other limb apraxic subtests, such as the imitation of meaningless gestures (Goldenberg and Randerath, 2015; Hoeren et al., 2014) or the actual use of tools (Goldenberg and Spatt, 2009; Martin et al., 2016; Salazar-López et al., 2016). Goldenberg (2009) proposed that an underlying function of the inferior parietal lobe is the categorical apprehension of spatial relationships including body part coding as well as the mechanical interaction between hand, tools and recipient object. Our results are in line with these accounts, which will be discussed in more detail later on.

#### 7.3.2 BPO strategy

Our results demonstrated that the pathological BPO use was associated with anterior lesions in inferior frontal as well as middle temporal regions extending to the temporal pole. Therefore, as hypothesized, BPO errors are mainly induced by anterior lesions of a left fronto-temporal network, which is assumed to be relevant for the retrieval and combination of semantic knowledge about tools and their use (Binder et al., 2009; Goldenberg and Spatt, 2009; Hogrefe et al., 2017; Tobia and Madan, 2017). Further, temporal pole lesions are related to semantic processing deficits (Hodges et al., 1992; McClelland and Rogers, 2003). The present neural findings are further underpinned by research on language processing that has already demonstrated the role of the posterior dorsal part of the temporal lobe and parietal operculum for the *translation* of acoustic speech signals into articulatory representations (Hickok and Poeppel, 2007), as well as the role of areas in the anterior inferior frontal cortex for semantic processes (Friederici and Gierhan, 2013). Transferred to research on limb apraxia, Nelissen et al. (2010) argued that these regions may also serve as an interface linking gesture recognition and gesture production. It is quite possible that these regions play a similar role in production of transitive gestures as well. Based on the evidence from the present and previous findings, we propose that BPO errors in the pantomime task result from lesions in a separate processing stream that is closely linked to semantic and communication processes.

### 7.4 Two core networks to pantomime

The present neural findings imply that there are two core properties on which pantomime production is based: 1) motor cognition that needs to deal with absence of mechanical interaction between the hand and objects, as well as the replication of motor programs of actual use; and 2) gestural communication, depicting objects and actions in a way that others can understand. Similar associations have been demonstrated when the gestures to be imitated have a meaning for the initiator, but not when the gestures to be imitated carry no meaning (Mengotti et al., 2013). This dissociation can be explained by the dual-pathway model of gesture imitation (Goldenberg, 1996; Rothi et al., 1991; modified by Tessari et al., 2007; Tessari and Rumiati, 2004). According to this model, new gesture imitation relies on a direct non-semantic pathway, responsible for the reproduction of the seen gestures. In contrast, imitation of familiar gestures relies on a semantic pathway, through which gestures are produced by accessing their meanings in the semantic memory.

**Fig. 4:**
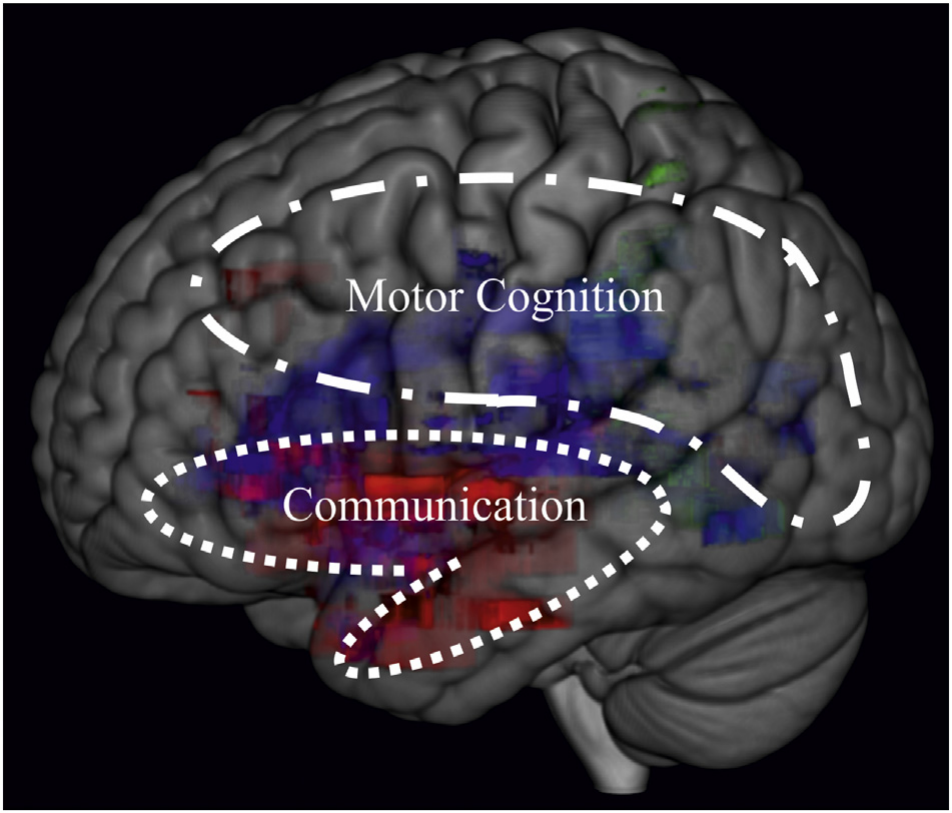
Model of two-core-networks for pantomime production. Based on the two core properties of pantomime production, motor cognition impaired by posterior fronto-parietal lesions and gestural communication impaired by lesions in more anterior fronto-temporal brain regions. (Colored areas are based on the statistical analysis considering grip errors (green), movement errors residuals (blue) and the use of a BPO strategy (red); presented in Fig. 2).

Based on the present results, we propose a working model of pantomime production with two core networks combining praxic and linguistic performance. The model integrates dual-stream accounts concerning action (Binkofski and Buxbaum, 2013) and language processing (Friederici and Gierhan, 2013; Hickok and Poeppel, 2007). Depending on the error-type the production of transitive pantomime gestures can be impaired either due to lesions predominantly in a *motor-cognitive network* in the dorsal action stream (movement and grip errors, blue) and/or due to damage in a *communicative network* including a ventral language stream that is related to semantic processing (BPO strategy, red; Fig. 4). Further, the shared regions of the two networks are demonstrated by overlap in more anterior areas, i.e. of the ventro-dorsal action stream or dorsal language stream regions, respectively. Together, the *motor-cognitive* and the *communicative network* comprise a large pantomime network that covers the reported prominent regions in a fronto-temporo-parietal territory. It is conceivable that the semantic-communicative aspects preponderate the more ventral-anterior the axes of the pantomime network gets, while the more dorsal-posterior involved areas are dominated by the spatial-configural character of motor cognition. The SMG may represent a key structure in an integrative hub (for a similar interpretation see e.g. Frey, 2007; Króliczak et al., 2016; Vingerhoets, 2014).

The proposed distinction between two core aspects of pantomime – motor-cognitive and a communicative – fits well with the findings and assumptions raised in a recent study by Goldenberg and Randerath (2015) that analyzed shared neural substrates of aphasia and apraxia. Overlapping lesion maps of pantomime and several linguistic tests of the Aachen Aphasia Test (naming, comprehension, repetition, written language and Token Test) were found in anterior temporal lesions. In contrast overlap of the pantomime lesion map in the inferior parietal lobe was only found for specific linguistic tests, namely written language and the Token Test, as well as the imitation of meaningless gestures. These tasks all have the apprehension of perceived categorical relationships of stimulus-properties in common (e.g. Written Language: features of letters; Token: color, shape, size; Imitation of meaningless postures: body-parts; Pantomime: body parts and the imagined tools and objects). The authors speculated that the coincidence of language impairment and defective pantomime in anterior regions is due to semantic memory and proposed that the overlap in parietal regions fits best with the categorical perception of (spatial) relationships. Further support for the specification of the communicative network in our proposed explanatory working model is provided by findings in the new study by Hogrefe et al. (2017), who tested the patients' ability to express video contents by gesturing. The resulting gestural narrations in turn were identified by raters (Identification Rates). Consistent with this interpretation of our current findings, their VLSM analysis suggested that the patients' poor gestural expression was associated with lesions in anterior temporal and inferior frontal regions. (Please see supplementary material Appendix C for a brief discussion of BPO errors and impairments in imitation of meaningful gestures and language production).

We propose a working model of two core networks for pantomiming the use of single tools that was purposely kept simple and that does not claim exclusivity. For instance, it is conceivable that there are mechanisms of motor cognition and communication that overlap, or that additional (i.e. rather frontal) lesion locations are crucially involved when pantomiming sequential actions. Despite its simplicity, there are some major advantages of our distinction between communicative and motor-cognitive aspects of pantomime. First, it provides an explanation for the current discrepancies between findings in lesion studies. Second, it is supported by our study results. Third, it goes along well with comorbid existing neuropsychological impairments, which for example are related to language or apraxia of actual tool use.

However, while the model provides a helpful explanation for the divergent results between studies, the dichotomy does not depict the general clinical picture. Aphasia highly correlates with apraxia. The occurrence of movement errors correlates highly with BPO errors. And, lesion size in general frequently correlates with the grade of impairment. All these points demonstrate that small focal lesions or discrete functional loss is atypical after stroke. Especially when tasks such as pantomiming tool use combine different error types or major functions, there is a high risk that the resulting lesion mapping data may become divergent.

## 8 Conclusion

With this work, we aimed to clarify discrepancies between findings concerning the neural correlates of pantomiming tool use. Motor-cognitive aspects of pantomime production including impaired grip and movement performance were associated with damage in posterior (rather dorsal) regions in the fronto-temporo-parietal pantomime network, whereas communicate aspects represented by a pathological use of BPO were linked to anterior (rather ventral) fronto-temporal lesions in this pantomime network.

With our working model of two core nodes in a pantomime network – a motor-cognitive and a communicative node based on the dual-route approaches - we aim to stimulate further research that may help to further disentangle the underlying mechanisms of pantomiming. For future studies that may test the proposed model for pantomime of tool use, we suggest considering both divergent and convergent assessments of motor cognition and communicative abilities, and to include specific measures of communicative gesture production.

## Acknowledgements & Funding

This work was supported by grants of the DFG (grant no. RA2492/3-1) and the EU FP7 Marie Curie Zukunftskolleg Incoming Fellowship Programme at the University of Konstanz (grant no. 291784) awarded to Dr. Jennifer Randerath.

## Appendix A–C Supplementary data

Supplementary material Image 1
