# Supplementary material for: It takes two to pantomime: Communication meets motor cognition
Source: Neuroimage Clin. 2018 Jun 21;19:1008–17. doi: 10.1016/j.nicl.2018.06.019 (PMC6039835; doi:10.1016/j.nicl.2018.06.019)
Supplement: Supplementary file 1 — Supplementary material [file mmc1.docx]

# **Appendix A**

**Supplementary Table 1: Raw scores and distribution of the total pantomime score in LBD patients. 73% of the LBD patients scored worse than the Cut-Off value of 34 points (indicated by bold line), demonstrating limb apraxia. Dark grey indicates cases performing below 50% of the Cut-off value (N=7, 10.5%) and light grey indicates cases performing below 75% of the Cut-off value (N=7, 10.5%).**

| **LBD patients** | **Total pantomime score** | **Frequency (N)** | **Percent**  **(N%)** |
| --- | --- | --- | --- |
|  | 8 | 1 | 1,5 |
|  | 9 | 1 | 1,5 |
|  | 11 | 1 | 1,5 |
|  | 12 | 1 | 1,5 |
|  | 13 | 1 | 1,5 |
|  | 17 | 2 | 3,0 |
|  | 20 | 1 | 1,5 |
|  | 21 | 1 | 1,5 |
|  | 23 | 2 | 3,0 |
|  | 24 | 2 | 3,0 |
|  | 25 | 1 | 1,5 |
|  | 26 | 5 | 7,5 |
|  | 27 | 4 | 6,0 |
|  | 28 | 2 | 3,0 |
|  | 29 | 2 | 3,0 |
|  | 30 | 3 | 4,5 |
|  | 31 | 3 | 4,5 |
|  | 32 | 6 | 9,0 |
|  | 33 | 10 | 14,9 |
|  | 34 | 7 | 10,4 |
|  | 35 | 8 | 11,9 |
|  | 36 | 3 | 4,5 |
|  | **Total** | **67** | **100,0** |

# **Appendix B**

**Additional Analysis for BPO-items**


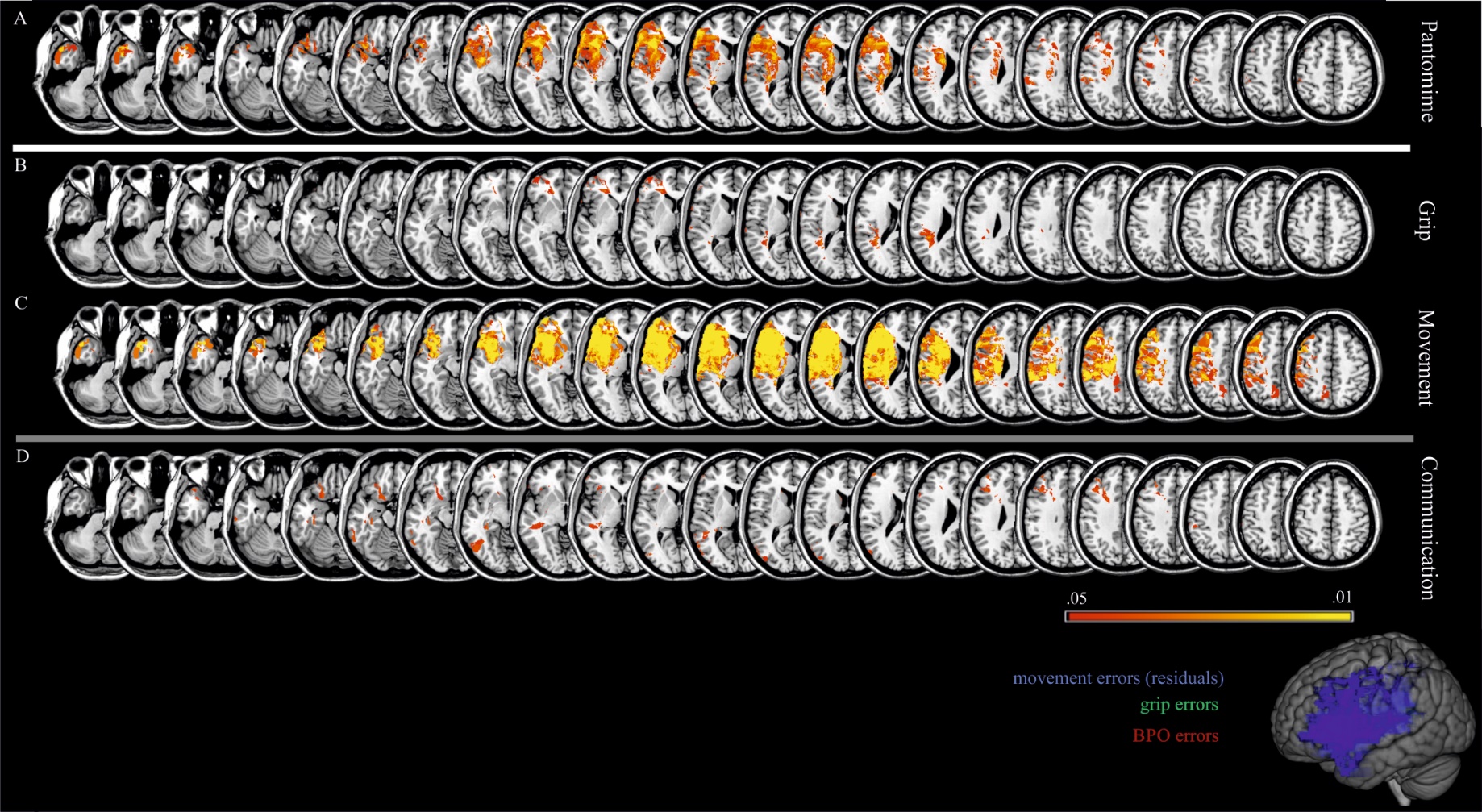


**Supplementary Fig. 1: VLSM analyses for error types limited to BPO items (t-test values, FDR corrected with p < .05; except for BPO errors).**

The first map (A) depicts lesion locations that are associated with an impaired pantomime production (total pantomime score). Following maps display voxels that are either associated with motor-cognitive errors in pantomime production (grip (B) and movement residuals (C)) or communicative errors (BPO errors (D)). Critical lesion areas associated with movement (blue), grip (green) and BPO errors (red) are additionally displayed in the sagittal view. Please note, that BPO errors and movement errors marginally correlate (r = -.189, r = .069) and we therefore utilized residuals. Further, BPO errors are not FDR corrected.

# **Appendix C**

**Supplementary Discussion. BPO errors and impairments in imitation of meaningful gestures and language production.**

Based on one reviewer’s suggestions we here consider other behavioral variables generally related to communication for a subgroup of patients (N=27) for whom this data was available (patients recruited at the Kliniken Schmieder in Allensbach). We provide correlational data for BPO errors as gestural pantomime errors on the one hand and the performance in imitating meaningful gestures as well as language production (AAT Naming) on the other hand. The following inconclusive results likely have to be attributed to the insufficient sample size and unspecificity of the present variables with respect to communicative gesture production.

There was no significant correlation between imitation of meaningful gestures and the frequency of BPO errors in No-BPO items (r = .027, p = .874) or BPO-items (r=-.132, p = .413). This might be attributable to the fact that the imitation of meaningful gestures can also be performed without using communicative strategies. Imitation of a perceived posture can be based on online processing of spatial relationships of body parts in space, whereby the understanding of the gesture’s meaning is not mandatory.

There is a significant correlation between language production deficits (AAT Naming) and more frequent movement errors (r = -.382, p = .013), grip errors (r = -.574, p < .001) but only a trend demonstrating a slight association with BPO errors in No-BPO items (r = -.217, p =.188) and BPO-items (r = -.253, p = .105). One likely reason for these rather unspecific correlations is that these abilities all tap into the same networks. Another explanation may be, that patients with language deficits (N=17) do have significantly larger lesions (U = 19.5, p < .001) compared to the non-aphasic group (N=10). This increases the likelihood for patients with aphasia to be also impaired in pantomiming tool use. From this data here, conclusions about the influence of language deficits on communicative aspects in pantomime gestures appear limited. A more suitable test would have been the one used in a recently published VLSM study by part of the co-authors: the comprehensibility of gestural narrations (Identification Rates) (Hogrefe K., Ziegler W., Weidinger N., & Goldenberg G. (2017). Comprehensibility and neural substrate of communicative gestures in severe aphasia. Brain and Language, 171, 62-71.). Their VLSM analysis suggested that poor gestural expression was associated with lesions in anterior temporal and inferior frontal regions, going along with our interpretation of our present imaging findings. In line with the aforementioned results, in the study by Hogrefe et al. there were no significant correlations of the Identification Rates with the subtest Naming and Token Test of the AAT as indicators for the severity of aphasia and verbal output.
